# Study of Two Sedative Protocols for Drug-Induced Sleep Endoscopy: Propofol versus Propofol-Remifentanil Combination, Delivered in Target-Controlled Infusion Mode

**DOI:** 10.3390/medicina60071123

**Published:** 2024-07-12

**Authors:** Narcis-Valentin Tănase, Răzvan Hainăroșie, Lăcrămioara-Aurelia Brîndușe, Cristian Cobilinschi, Madalina Dutu, Dan Corneci, Viorel Zainea

**Affiliations:** 1 Department of Anaesthesia and Intensive Care Medicine, Carol Davila University of Medicine and Pharmacy, 050474 Bucharest, Romania; cristian.cobilinschi@umfcd.ro (C.C.); madalina.dutu@umfcd.ro (M.D.); dan.corneci@umfcd.ro (D.C.); 2 Clinic of Anaesthesia and Intensive Care Medicine, Dr. Carol Davila Central University and Emergency Military Hospital, 010825 Bucharest, Romania; 3 Department of Ear, Nose and Throat, Carol Davila University of Medicine and Pharmacy, 050474 Bucharest, Romania; razvan.hainarosie@umfcd.ro (R.H.); viorel.zainea@umfcd.ro (V.Z.); 4 Department of Public Health and Management, Carol Davila University of Medicine and Pharmacy, 050463 Bucharest, Romania; 5 Clinic of Anesthesiology and Intensive Care, Clinical Emergency Hospital, 014461 Bucharest, Romania

**Keywords:** obstructive sleep apnea (OSA), drug-induced sleep endoscopy (DISE), remifentanil, propofol, target-controlled infusion (TCI), sedation, bispectral index (BIS), airway dynamics, pharmacologically induced sleep, sedation protocols

## Abstract

*Background and Objectives:* Obstructive sleep apnea (OSA) is a prevalent sleep-disordered breathing pathology with significant clinical consequences, including increased cardiovascular risk and cognitive decline. Continuous positive airway pressure (CPAP) is the gold-standard treatment, but alternative strategies are sometimes needed for patients intolerant to CPAP. Drug-induced sleep endoscopy (DISE) is a key diagnostic tool for assessing upper airway obstruction in OSA patients and subsequently tailoring a surgical approach, with sedation protocols playing a crucial role in its efficacy and results accuracy. This study aimed to investigate the effect of adding remifentanil to a propofol target-controlled infusion (TCI) regimen on the sedation parameters and procedural outcomes of DISE. *Materials and Methods:* The study was conducted at the Central University and Emergency Military Hospital “Dr. Carol Davila” and Ria Clinic in Bucharest between July 2021 and October 2023. Thirty-one patients were enrolled and randomised into two groups: a propofol group (P group, *n*= 11) and a remifentanil-propofol group (R-P group, *n* = 20). DISE was performed using standardised protocols, sedative drugs were administered in TCI mode, and data on sedation levels, respiratory and cardiovascular parameters, and procedural incidents were collected. *Results:* The addition of remifentanil at 1 ng/mL effect-site concentration significantly reduced the effect-site concentration of propofol required for adequate sedation (3.4 ± 0.7 µg/mL in the P group vs. 2.8 ± 0.6 µg/mL in the R-P group, *p* = 0.035). The time to achieve adequate sedation was also shorter in the R-P group (7.1 ± 2.5 min vs. 9.5 ± 2.7 min, *p* = 0.017). The incidence of cough, hypoxemia, and cardiovascular events did not significantly differ between the two groups. *Conclusions:* Adding remifentanil to a propofol TCI regimen for DISE effectively reduces the required propofol effect-site concentration and shortens sedation time without increasing the risk of adverse events. This combination may enhance the safety and efficiency of DISE, offering a promising alternative for patients undergoing this procedure.

## 1. Introduction

Obstructive sleep apnea (OSA) represents one of the most frequent sleep-disordered breathing pathologies, characterized by repetitive episodes of partial or complete obstruction of the upper airway, resulting in iterative breathing pauses, subsequent hypoxemia, and disturbed sleep architecture [1]. A recent study analyzing data from 16 countries concerning the global prevalence of OSA revealed nearly 1 billion people are affected, with the prevalence surpassing 50% in some countries [2]. The global prevalence is estimated to be approximately 22.6% [3]. Common risk factors of OSA include obesity, advancing age, male gender, smoking and alcohol use, neck circumference, family history, and some medical conditions (acromegaly, hypothyroidism, and goiter independently of thyroid function) [3,4,5]. Clinical consequences of OSA are increased cardiovascular risk, arterial hypertension, cognitive decline, obesity, and elevated risk of being part of a vehicle accident [6]. Continuous positive airway pressure (CPAP) represents the cornerstone of OSA treatment. Patients who are intolerant to CPAP therapy can become candidates for alternative treatment strategies, such as different site surgeries, oral appliances, hypoglossal stimulation techniques, myofunctional therapy, or behavioural changes. Phenotypic subtypes of OSA have recently been described [7] in this heterogeneous condition; therefore, the importance of a personalized approach in the diagnosis and therapeutic management of the disease is emphasized.

Consequently, accurately revealing the sites of an upper airway obstruction and its dynamic represents a cornerstone in tailoring personalized treatment approaches for OSA patients. The standard diagnostic tool in this setting consists of awake fiberoptic nasopharyngeal endoscopy [8], usually in conjunction with the Muller manoeuvre. This manoeuvre consists of attempts to inhale with the mouth closed and the nostrils plugged, which leads to a collapse of the airway. The examiner may witness the collapse and identify weakened airway segments by introducing a flexible endoscope into the hypopharynx to obtain a view. However, this investigation offers exclusively static information regarding the upper airway [9]. 

A drug-induced sleep endoscopy (DISE) supplementary provides a comprehensive assessment of the upper airway’s anatomy and dynamics (obstruction pattern) during pharmacologically reproduced sleep. DISE was proposed for the first time in 1991 by Croft and Pringle [9] as an adjunctive tool aiding in assessing airway dynamics during sleep. Evaluation of the upper respiratory airway in patients with sleep-disordered breathing using DISE has become the preferred instrument in the diagnostic approach to this disease. This evaluation aims to accurately reproduce the state of natural sleep in patients with snoring and obstructive sleep apnea. The surgeon explores the airway using a flexible fiberscope once the hypnosis state reaches the desired level and the patient demonstrates snoring and apneic events. With an appropriate sedation technique, the investigator performing the endoscopy can adequately identify and differentiate the site, the grade, and the dynamic pattern of the obstruction. Assessing airway collapse presents a paramount importance in tailoring appropriate and personalized surgical treatment in patients not tolerating CPAP.

The protocol for the DISE technique has not been standardised until now. The intravenous anesthetic drugs most frequently used for DISE are midazolam and propofol [10]. These drugs present some pharmacologic properties that confer advantages in achieving the desired level of sedation. However, propofol sedation for DISE may be associated with an increased risk of airway collapsibility. The mechanism of increasing airway collapsibility involves a dose-dependent inhibition of genioglossus muscle activity that seems to be induced by the association between depression of the upper airway reflexes and of the central respiratory output to the dilator muscle of the airway [11]. Consequently, performing DISE at the lowest effect-site concentration of propofol would confer the benefit of mitigating the sedative drug effect on the upper airway during pharmacologically induced sleep. Recent research suggests that remifentanil may offer advantages as an adjunctive sedative drug by decreasing the cough reflex, shortening the procedure time, and decreasing the effect-site concentration required for propofol sedation [12,13]. Adding opioids to sedative protocols of DISE remains a debatable topic. Controversies arise from opioids’ impact on respiratory depression, increasing the level of sedation and interference with overall diagnosis accuracy. The optimal remifentanil concentration necessary for lowering the propofol concentration for DISE has not been determined until now. 

In our study, we aimed to investigate the effect of supplementing a propofol sedation regimen administrated in target-controlled infusion (TCI) mode with the addition of remifentanil, also infused in TCI mode. Our primary outcome was to study the effect of adding remifentanil on the effect-site concentration of propofol administered using the Schnider model at the point of adequate sedation for fibroscopy. Secondary outcomes consisted of evaluating the incidence of respiratory depression during the procedure; the level of sedation depth, objectively assessed using the bispectral index (BIS), sneezing, and cough reflex; the time required up to the moment of starting the procedure; and the incidence of cardiovascular adverse events (hypotension, bradycardia).

## 2. Materials and Methods

The study was accomplished at the Central University and Emergency Military Hospital “Dr. Carol Davila” and Ria Clinic Cotroceni in Bucharest between July 2021 and October 2023. The study was approved by the Institutional Ethics Committee. The study was registered in the Research Registry with the unique identifying number researchregistry10409. After obtaining the ethics committee’s approval, the patients signed the informed consent for participating in the study. We enrolled 31 patients proposed for DISE, The American Society of Anesthesiologists (ASA) physical status class I-II, aged between 18 and 70 at the moment of inclusion in the study. We excluded from the study patients with chronic cardiac pathologies (congestive cardiac failure and ischemic heart disease), chronic obstructive pulmonary disease, asthma, chronic kidney disease, chronic liver failure, alcohol use disorder, psychiatric disease, or a body mass index (BMI) > 40 kg/m^2^ and an allergy to propofol or remifentanil. All patients had a detailed medical history and detailed pre-investigation ear, nose, and throat (ENT) examination, consisting of a naso-endoscopy with the Muller manoeuver and a polysomnography (PSG). All subjects presented a documented diagnosis of OSA with an apnea-hypopnea index (AHI) of >15 registered via the polysomnography anterior to enrolling in the study. All patients presented an indication of alternative treatment for OSA, due either to documented intolerance to CPAP therapy or CPAP refusal. We also recorded from the preprocedural PSG the minimum SpO2 during natural sleep for every patient. We enrolled consecutively all patients presenting inclusion criteria, using a 2:1 randomization algorithm. 

The patients were randomly distributed to the propofol group (P group, *n* = 11) and remifentanil-propofol group (R-P group, *n* = 20). DISE was performed in the operating room. Premedication was not administered. We did not use any preparatory measures such as topical local anesthesia, nasal decongestion, and antisecretory drugs.

The patient’s positioning involved lying in a supine position with a pillow under their head. The head was maintained in a neutral position during the examination. All investigations were performed maintaining a comfortable atmosphere for the patient, with silence and low-intensity ambient lighting. 

An intravenous cannula was inserted into a peripheral vein of the left arm and standard monitoring of the ECG, non-invasive blood pressure, and pulse oximetry (multiparameter monitoring system Dräger Infinity Delta). Additionally, to objectively assess the sedation level, a BIS monitor (Aspect Medical Systems, Inc., Norwood, MA, USA) was attached. After obtaining a baseline BIS registration, we connected the TCI pumps to the patient’s intravenous cannula through an octopus-type connector.

### 2.1. Sedation Procedure

The sedation in the P group was performed using a target-controlled infusion (TCI) system pump (BBraun, Melsungen, Germany) using 1% propofol with an initial target concentration of 1 µg/mL with the effect-site targeted concentration in the Schnider model. The target concentration was gradually increased in a stepwise manner with 0.5 µg/mL every 2 min, carefully observing the clinical sedation status. The target was to establish a level of sedation between BIS 70 and 50, the patient clinically demonstrated cycles of apnea–hypopnea, and the otolaryngologist was able to perform the procedure. Sedation was performed for all patients by the same consultant anesthesiologist (NVT), with 20 years of experience in a teaching hospital in the field of clinical anesthesia (including experience in total intravenous anesthesia and TCI).

In the R-P group, remifentanil infusion was commenced first, at a target effect concentration of 1 ng/mL using the Minto effect-site model with the same type of system pump. This remifentanil concentration was maintained unchanged throughout the entire investigation. After 2 min from starting remifentanil, propofol was also commenced, following the same prescribing dosage as group P. We did not routinely apply supplemental oxygen during DISE. All endoscopies were performed by the same experienced ENT surgeon (RH) with additional expertise in sleep medicine, assisted by at least one other specialist ENT surgeon. Patients were transferred to the recovery unit for further monitoring after the investigation.

We recorded data regarding baseline cardiovascular status (vital parameters), sedation state, using the bispectral index, the moment of performing the endoscopy from the start of the sedative procedure (the time to achieve adequate sedation), incidence of respiratory depression (defined as SpO_2_ < 90%), procedural occurrence of sneezing or cough, and cardiovascular adverse events (hypotension, bradycardia). Adverse events and outcomes were recorded by an independent investigator who was blinded to the treatment assignments. All data related to adverse events and outcomes were anonymized before analysis. For classification purposes, the VOTE system (velum, oropharynx, tongue base, epiglottis) was used [10]. We registered the occurrence of arterial hypotension as systolic blood pressure < 90 mmHg and bradycardia as HR < 50/min).

### 2.2. Data Analysis

Data analysis was performed with SPSS (Statistical Package for Social Sciences) Version 29.0 software (IBM SPSS Statistics for Windows, Version 29.0. Armonk, NY, USA: IBM Corp). We analyzed the distribution of continuous variables, and we presented the means and standard deviations. The *t*-test was used to compare the means of continuous variables by group (P and R-P groups). The qualitative data were presented as counts and percentages. We used the chi-square test or Fisher’s exact test to compare the distribution of the categorical variables between the P group and the R-P group. A *p*-value < 0.05 was considered statistically significant.

## 3. Results

We enrolled a total of 31 patients in the study: 11 in the propofol group (group P) and 20 in the remifentanil-propofol group (group R-P). All patients accomplished the endoscopy, without any complications. We conducted the statistical analysis using the same number of patients that we enrolled in the study, for both groups. The characteristics of the patients are presented in Table 1. These characteristics were not significantly different between the P and R-P group. 

The descriptive characteristics of the patients, by group, are presented in Table 1. There are no significant differences in average age, height, weight, and body mass index. Also, there are no significant differences in sex, ponderal status, and ASA risk distribution.

The severity of OSA quantified by the average apnea-hypopnea index (AHI index), lowest SpO_2_ during natural sleep, and smoking prevalence are similar in both groups. Also, the prevalence of arterial hypertension as a comorbidity is not different between groups (Table 1). 

Table 2 shows the main parameters registered during DISE. The mean value of the effect-site concentration of propofol established at the initiation of the endoscopy in group P (3.4 ± 0.7 µg/mL) was significantly higher (*p* = 0.035) than in group R-P (2.8 ± 0.6 µg/mL). 

The period until achieving adequate sedation from starting the sedative infusion to successfully inserting the endoscope and proceeding to examination was 9.5 ± 2.7 min in group P, significantly longer (*p* = 0.017) than in group R-P (7.1 ± 2.5 min). 

The lowest oxygen saturation registered during DISE, calculated as mean values, did not statistically differ between groups (89.5 ± 6.9 in group P and 84.8 ± 6.6 in group R-P, *p* = 0.073). Baseline BIS (BIS at the start of the sedation) was similar in the two groups (*p* = 0.486). The sedation level achieved at the moment of starting the endoscopy did also not register differences between the two groups (69.3 ± 5.9 vs. 68.9 ± 6.1, *p* = 0.919). Preprocedural oxygenation did also not differ significantly between groups (*p* = 0.072). 

Table 3 illustrates the incidence of cough during the procedure and the occurrence of cardiovascular incidents (hypotension and bradycardia) in the two groups. We recorded the occurrence of cough in 3 patients in group P (27.3%) and in 4 patients (20%) in group R-P; this difference was not statistically significant (*p* = 0.484). In group P, one patient required supplemental oxygen administration due to severe hypoxemia, and in group R-P, three patients required oxygen (without significant difference between groups, *p* = 0.553). 

The results of DISE, consisting of grade and dynamic pattern of airway obstruction, were quantified using the VOTE classification (velum, oropharynx, tongue base, epiglottis). The results are summarized in Table 4.

## 4. Discussion

We studied the efficacy and safety of adding remifentanil to a propofol TCI sedation regimen for DISE. Multiple questions have arisen in the literature concerning which drug would be ideal in performing DISE and the adequate level of sedation for which the airway endoscopy should be undertaken to accurately reproduce events from normal sleep and consequently allow an optimal surgical decision. 

We evaluated in this study two approaches in performing sedation: propofol delivered in TCI mode, using the Schnider model adjusted to effect compartment, and a combination of propofol and remifentanil TCI administered using the Minto model in effect-site titration. TCI sedation provides the advantage of delivering a more stable and controlled level of sedation because increasing propofol concentration is associated with higher pharyngeal muscle relaxation and collapsibility [11] in a dose-dependent manner.

DISE under TCI is suggested as the first choice in evaluating the obstruction pattern of the upper airway. Although, generally, the addition of co-sedatives such as opiates to the quasi-unanimously accepted propofol- or midazolam-based DISE sedative regimen is controversial [8], our purpose was to investigate the role of adding remifentanil in a reduced concentration to a sedative TCI propofol regimen. In our study, the mean effect site concentration of propofol necessary for performing DISE in the P group was 3.4 µg/mL, comparable to the values obtained in two other previous studies (3.04 µg/mL, 3.39 µg/mL) [12,13].

The addition of remifentanil to propofol was previously evaluated [12,13], but in a different effect-site concentration than our study (1 ng/mL and respectively 1.5 ng/mL). In concordance with data published by Kim et al. [13], we demonstrated a reduction of the effect-site concentration of propofol delivered in TCI with the Schnider model via the addition of a remifentanil 1 ng/mL effect-site. The Schnider model was primarily developed using data from non-obese patients [14], which raises concerns about its applicability in obese populations. Obese patients have different pharmacokinetic profiles due to factors like increased fat mass, altered blood flow, and changes in the volume of distribution. These differences can affect drug distribution, metabolism, and clearance [15].

Regarding the protocol of propofol TCI administration in our study, and to overcome the intrinsic inadvertencies of the Schnider model applied in our mostly obese patients, we decided to set a lower than recommended initial target concentration in the effect compartment (1 µg/mL effect-site) and subsequently increase concentration by 0.5 µg/mL every 2 min, aiming for a very smooth induction, increasing the safety of the examination and decreasing the risk of oversedation and airway inadvertent obstruction, non-related to sleep breathing disorder. Moreover, this slow increase in effect-site concentration of propofol allowed us to obtain an accurate and sufficient observation window. 

Our results indicate that the addition of remifentanil in this study into a drug-induced sleep endoscopy (DISE) target-controlled infusion (TCI) propofol regimen significantly reduces the required effect-site concentration of propofol to achieve adequate sedation for DISE. Significantly, this adjustment does not correlate with an increased risk of hypoxemia. The incidence of oxygen desaturation did not significantly differ between the two groups (*p* = 0.072); however, we observed a tendency to lower SpO2 saturation in the P-R group (84.8%) compared to the P group (89.5%).

Furthermore, these findings suggest that the synergistic use of remifentanil with propofol may enhance the safety and efficacy of DISE procedures by optimizing sedation levels while maintaining respiratory stability. In contrast with results published by Cho et al. [12], who studied a similar pattern of sedation in DISE by adding remifentanil at 1.5 ng/mL, we did not objectivate significant desaturation using the protocol with remifentanil administered at 1 ng/mL TCI. The addition of remifentanil to an effect-site TCI propofol sedation regimen also resulted in a statistically significant decrease in the time to start the endoscopic procedure (time to adequate sedation), 7.1 ± 2.5 min vs. 9.5 ± 2.7 min, *p* = 0.017. This addition of remifentanil to the propofol sedation regimen did not demonstrate a significant impact on vital signs and upper airway dynamics during DISE compared to propofol alone. 

We surprisingly found that the incidence of cough during the endoscopic examination was not significantly different between the two groups; this result attests that adding the remifentanil at 1 ng/mL does not significantly facilitate the tolerance of the DISE procedure. In contrast, one previous study [12] demonstrated the complete absence of cough in 22 patients in the group sedated with propofol-remifentanil (remifentanil at an effect-site concentration of 1.5 µg/mL) but with significantly higher oxygen desaturation. With the reserve of a limited number of patients in our study, we have observed only a tendency to decrease the incidence of cough in the group R-P (20% vs. 27.3%) and improve the tolerance of endoscopy, without statistical significance (*p* = 0.484).

The sedation level at the start of the endoscopy, quantified via BIS monitoring, did not register a significant difference between the two groups (69.2 in group P vs. 68.9 in group R-P). These values are consistent with other published literature data [16]. The quality of sedation, as measured using the bispectral index (BIS), was similar between the two groups, indicating that both regimens provided an adequate depth of sedation. Interestingly, the time to achieve adequate sedation was significantly shorter in the R-P group, suggesting that the addition of remifentanil may facilitate a more rapid initiation of the endoscopic procedure. 

The endoscopic findings quantified by the VOTE classification system used to evaluate the upper airway obstruction patterns showed no significant differences between the two groups. Apparently, this consistency implies that adding remifentanil does not alter the diagnostic outcomes of DISE, further supporting its use as an adjunct in these procedures. Nevertheless, we prudently present this comparison regarding the VOTE findings in the two groups; these findings could also be influenced by the heterogeneity in OSA phenotypes, despite the similar characteristics of the patients, as illustrated by Table 1, and independent of the sedative protocol. 

This study has several limitations. On the first hand, the majority of enrolled patients were obese and the selection of the Schnider model in this subset of patients may not be the optimal choice due to the previously mentioned complex alteration in the pharmacokinetic-pharmacodynamic (PK/PD) profile in these patients. To overcome this interference, we chose to start the procedure with a low effect-site concentration and gradually increase the target while carefully monitoring the patient’s sedation state, improving the safety of the examination and obtaining sufficient observation time. Another limitation of the study resides in enrolling a limited number of patients. The relatively small sample size and the inclusion of specific patient populations (e.g., those with obesity) limit the generalizability of our findings. Additionally, while the Schnider model was used for propofol TCI, its applicability in obese patients can be problematic due to altered pharmacokinetic profiles. Future studies with larger and more diverse populations are needed to validate our findings and refine sedation protocols for DISE.

## 5. Conclusions

In conclusion, our study provides insights into the sedation strategies for drug-induced sleep endoscopy (DISE) in patients with obstructive sleep apnea (OSA). We demonstrated that the addition of remifentanil TCI to a propofol target-controlled infusion regimen effectively reduces the required effect-site concentration of propofol without significantly increasing the risk of hypoxemia or other adverse events. This synergistic pharmacokinetic approach not only maintains adequate sedation levels but also shortens the time to achieve sedation, potentially enhancing the efficiency and safety of DISE procedures.

Our findings suggest that remifentanil, when used at an effect-site concentration of 1 ng/mL, offers, through a beneficial pharmacokinetic synergism, the opportunity to reduce propofol dosage requirements, shortening the time for starting the endoscopic procedure. However, it does not significantly impact the incidence of cough or other complications compared to propofol alone. These results support the potential of a combined remifentanil-propofol regimen as a viable and possibly superior alternative to propofol TCI monotherapy for DISE.

Despite the promising outcomes, the study’s limitations, including the small sample size and the predominance of obese patients, warrant caution in generalizing the results. Future research with larger, more diverse populations and refined protocols is necessary to validate and expand upon our findings.

Ultimately, our study underscores the importance of personalized and carefully monitored sedation strategies in optimizing DISE for patients with OSA, paving the way for improved diagnostic accuracy and tailored therapeutic interventions. 

## Figures and Tables

**Table 1:** Characteristics of patients by group.

Characteristic	Group P * (*n* = 11)	Group R-P ** (*n* = 20)	*p*-Value
Age (year) (mean ± SD)	46.5 ± 7.9	48.4 ± 11.8	0.638
Sex			0.445
Male (%)	11 (100.0)	19 (95.0)	
Female (%)	0 (0.0)	1 (5.0)	
Height (cm) (mean ± SD)	177.5 ± 6.2	176.1 ± 6.8	0.587
Weight (kg) (mean ± SD)	94.5 ± 13.2	97.5 ± 16.9	0.621
Body mass index (kg/m^2^) (mean ± SD)	30.0 ± 3.8	31.4 ± 5.2	0.427
Ponderal status			0.995
Normoponderal (BMI 18.5–24.9 kg/m^2^)	1 (9.1)	2 (10.0)	
Overweight (BMI 25–29.9 kg/m^2^)	4 (36.4)	7 (35.0)	
Obese (BMI > 30 kg/m^2^)	6 (54.5)	11(55.0)	
ASA physical status l/ll	5 (45.5)/6 (54.5)	8 (40.0)/12 (60.0)	0.768
Apnea-hypoxia index in PSG (mean ± SD)	40.7 ± 19.3	47.8 ± 23.2	0.399
Hypertension (%)	4 (36.4)	6 (30.0)	0.717
Smoking (%)	2 (18.2)	4 (20.0)	0.902
Lowest SpO2 in normal sleep (%) (mean ± SD)	78.8 ± 6.8	79.9 ± 7.5	0.694

* group P—received propofol. ** group R-P—received remifentanil and propofol.

**Table 2:** Parameters registered during DISE.

Characteristic during DISE	Group P (*n* = 11)	Group 2 R-P (*n* = 20)	*p*-Value
Success (%)	11 (100.0)	20 (100.0)	1.00
Time (min) (mean ± SD)	9.5 ± 2.7	7.1 ± 2.5	0.017
Lowest SpO_2_ during DISE (%)	89.5 ± 6.9	84.8 ± 6.6	0.073
Preprocedural SpO_2_ (mean ± SD)	98.4 ± 1.2	97.2 ± 1.9	0.072
CeP (mean ± SD) *	3.4 ± 0.7	2.8 ± 0.6	0.035
BIS at starting endoscopy (mean ± SD)	69.2 ± 5.9	68.9 ± 6.1	0.919
BIS baseline (mean ± SD)	97.1 ± 1.4	96.7 ± 1.5	0.486

* = effect-site concentration of propofol.

**Table 3:** Incidents recorded during drug-induced sleep endoscopy by group.

Characteristic	Group P (*n* = 11)	Group R-P (*n* = 20)	*p*-Value
Cough (%)	3 (27.3)	4 (20.0)	0.484
Supplemental oxygen (%)	1 (9.1)	3 (15.0)	0.553
Hypotension	1 (9.1)	2 (10.0)	0.719
Bradycardia	0 (0.0)	2(10.0)	0.409

**Table 4:** Endoscopic findings, described with the VOTE classification.

Characteristics of Obstruction	P Group (*n* = 11)	R-P Group (*n* = 20)	*p*-Value
Velum			
Anteroposterior (partial/complete)	2 (18.2)/2 (18.2)	1 (5.0)/3 (15.0)	0.455
Lateral (partial/complete)	0 (0.0)/0 (0.0)	0 (0.0)/0 (0.0)	-
Concentric (partial/complete)	2(18.2)/3 (27.3)	3(15.0)/7 (35.0)	0.903
Oropharynx			
Lateral (partial/complete)	4 (36.4)/2 (18.2)	7 (35.0)/0 (0.0)	0.128
Tongue base			
Anteroposterior (partial/complete)	3 (33.3)/0 (0.0)	4 (28.6)/0 (0.0)	0.809
Epiglottis			
Anteroposterior (partial/complete)	1 (9.09)/1 (9.09)	4 (28.6)/3 (15.0)	0.211
Lateral (partial/complete)	2 (18.2)/0 (0.0)	3 (15.0)/1 (5.0)	

## Data Availability

Data is unavailable due to privacy restrictions.
